# The Physical and Mental Health of Head Start Staff: The Pennsylvania Head Start Staff Wellness Survey, 2012

**DOI:** 10.5888/pcd10.130171

**Published:** 2013-10-31

**Authors:** Robert C. Whitaker, Brandon D. Becker, Allison N. Herman, Rachel A. Gooze

**Affiliations:** Author Affiliations: Brandon D. Becker, Allison N. Herman, Rachel A. Gooze, Temple University, Philadelphia, Pennsylvania. Ms Herman is now at the Children’s Hospital of Philadelphia, Philadelphia, Pennsylvania. Dr Gooze is now at Child Trends, Bethesda, Maryland.

## Abstract

**Introduction:**

Despite attention to the health of low-income children in Head Start, little is known about the health of adults working for the program. The objective of our study was to compare the physical and mental health of women working in Pennsylvania Head Start programs with the health of US women who have similar sociodemographic characteristics.

**Methods:**

We used data from a web-based survey in 2012 in which 2,199 of 3,375 (65.2%) staff in 66 Pennsylvania Head Start programs participated. For the 2,122 female respondents, we determined the prevalence of fair or poor health status, frequent (≥14 d/mo) unhealthy days, frequent (≥10 d/y) work absences due to illness, diagnosed depression, and 3 or more of 6 physical health conditions. We compared these prevalences with those found in 2 national samples of employed women of similar age, education, race/ethnicity, and marital status.

**Results:**

Among Head Start staff, 85.7% were non-Hispanic white, 62.4% were married, and 60.3% had completed college. The prevalence (% [95% confidence interval]) of several health indicators was higher in Head Start staff than in the national samples: fair or poor health (14.6% [13.1%–16.1%] vs 5.1% [4.5%–5.6%]), frequent unhealthy days (28.3% [26.3%–30.2%] vs 14.5% [14.1%–14.9%]), diagnosed depression (23.5% [21.7%–25.3%] vs 17.6% [17.1%–18.0%]), and 3 or more physical health conditions (21.8% [20.0%–23.6%] vs 12.6% [11.7%–13.5%]).

**Conclusion:**

Women working with children in Head Start programs have poorer physical and mental health than do US women who have similar sociodemographic characteristics.

## Introduction

Head Start is the largest federally funded early childhood education program in the United States. Including Early Head Start, which serves children younger than 36 months of age, Head Start reaches nearly 1 million low-income children and their families and employs a staff of more than 200,000, most of whom are women. Staff include managers, classroom teachers, and home-based visitors, along with family service workers, who act as the primary liaison between programs and families. These staff are the key to achieving Head Start’s overarching goal of promoting school readiness by addressing children’s cognitive, social, emotional, and physical development and health.

For the staff to function well in their work with children and families, they must be well. However, Head Start staff have relatively low wages and social standing and often work under stressful circumstances. Many children in Head Start have multiple social risks, which can contribute to poor self-regulation and disruptive behaviors that interfere with learning and place emotional demands on staff (1). The stress experienced by staff could adversely affect their physical and mental health (2), which in turn could make it harder for staff to serve as models and meet the needs of children and families.

Despite the potential effect of stress on staff’s health and children’s outcomes in Head Start, we are not aware of any studies that describe the health of Head Start staff. The objective of this study was to determine the prevalence of physical and mental health conditions, poor health-related quality of life, and health care access in a defined population of Head Start and Early Head Start staff. We also compared the prevalence of these health indicators with the prevalence found in 2 national samples of adults who have similar sociodemographic characteristics.

## Methods

The Pennsylvania Head Start Staff Wellness Survey, conducted during 4 months in spring 2012, was a web-based survey of all staff working in the state’s Head Start and Early Head Start programs. Its purpose was to describe the physical health and psychological well-being of the staff and how their health and well-being relates to their functioning at work. The survey was intended to reach the entire staff, including program directors, managers, classroom teachers, home-based visitors, and family service workers. To minimize social desirability bias and ensure confidentiality, the survey was anonymous and voluntary. The research was approved by Temple University’s institutional review board.

### Participants

All 91 Head Start and Early Head Start programs in Pennsylvania were invited to participate in the survey. The Pennsylvania Head Start Association, a nonprofit advocacy organization for the state’s Head Start and Early Head Start programs and families, helped recruit programs to participate in the survey but was not involved in analyzing the data or reporting the findings. Of the 91 programs, 66 (72.5%) agreed to participate — 37 of 54 (68.5%) Head Start programs and 29 of 37 (78.4%) Early Head Start programs. Eighty percent of nonparticipating programs and 59.1% of participating programs were in metropolitan areas. From the Office of Head Start’s 2011 Program Information Report, which contains data on Head Start and Early Head Start staff aggregated at the program level, we obtained a count of the number of staff in each program. Each participating program director confirmed these numbers before the survey was administered. Directors also reported the number of managers on staff, data which were not available in the Program Information Report. From the 66 participating programs, 2,199 of 3,375 (65.2%) staff responded to the survey. The education levels and racial/ethnic composition of all staff in the 66 programs were similar to those of the subset of survey respondents.

### Survey development

The survey contained questions on health, psychological well-being, perceived stress, functioning at work, and sociodemographics. This report focuses on health and sociodemographics. These items were modeled on similar items in either the National Health Interview Survey (NHIS) (3) or the Behavioral Risk Factor Surveillance System (BRFSS) (4), allowing for comparison of the findings from our survey with the findings of these national surveys. Our web-based survey was created and hosted by using Qualtrics online survey software (Qualtrics Labs, Inc, Provo, Utah). After pretesting the survey with 11 Head Start staff, we shortened the survey so that it could be completed in 30 to 40 minutes.

### Survey administration

The director of each participating program was responsible for inviting all staff in his or her program to complete the survey, either at or away from work. The research team provided program directors with the online location of the survey website, printed materials to announce and describe the survey, and periodic updates on the response rates of all programs. The research team made no other contact with program staff and offered no monetary incentives to individual staff to participate in the survey. However, programs with a final response rate of at least 75% were entered into a raffle for 1 of 6 gift cards ranging in value from $100 to $250. Upon reaching the survey website, each respondent was first required to read a consent form, which explained that only aggregated data would be reported.

### Measures

We computed the prevalence of health indicators in 3 areas: physical and mental health, health-related quality of life, and health care access. The indicators were assessed with questions used in the 2011 version of either NHIS (3) or BRFSS (4).

**Physical and mental health:** Participants were asked about 6 physical health conditions associated with stress: obesity (5), asthma (6), hypertension (7), diabetes/prediabetes (8), severe headache/migraine (9), and lower back pain (10). Obesity (body mass index ≥30 kg/m^2^) was determined from self-reported height and weight; pregnant women were asked to give their prepregnancy weight. In separate yes/no questions, participants were asked if they had “ever been told by a doctor or other health professional” that they had asthma; hypertension or high blood pressure; diabetes or sugar diabetes (other than during pregnancy); and prediabetes or borderline diabetes. Finally, participants were asked separately whether (yes/no), during the previous 3 months, they had “severe headache or migraine that lasted a whole day or more” and “lower back pain that lasted a whole day or more.” We determined the prevalence of each of the 6 physical health conditions and of having 3 or more of these conditions.

Participants were asked (yes/no) if they had “ever been told by a doctor or other health professional” that they had depression. Although the 20-item Center for Epidemiologic Studies Depression Scale (CES-D) (11) is not part of NHIS or BRFSS, we asked participants to complete the CES-D, and we classified those with a score of 16 or more as depressed (11,12).

**Health-related quality of life:** Participants were classified as having fair or poor health status according to responses to the question, “Would you say your health in general is poor, fair, good, very good or excellent?” The prevalence of frequent physically unhealthy days, defined as 14 or more physically unhealthy days per month, was based on responses to the question (13), “Thinking about your physical health, which includes physical illness and injury, for how many days during the past 30 days was your physical health not good?” Similarly, the prevalence of frequent mentally unhealthy days, defined as 14 or more mentally unhealthy days per month, was based on the question (13), “Thinking about your mental health, which includes stress, depression, and problems with emotions, for how many days during the past 30 days was your mental health not good?” Finally, the prevalence of 10 or more work absences per year due to illness was based on the following, “During the past 12 months, about how many days did you miss work because of your own illness or injury (do not include maternity leave)?”

**Health care access**: Participants responded to 3 yes/no questions: “Do you have a personal doctor?” “Have you visited a dentist in the last 12 months?” and “Do you have health insurance coverage?”

### Data analysis

To facilitate a comparison of the prevalence of health indicators between our survey participants and the national samples from NHIS and BRFSS, we analyzed data only for women (n = 2,122). We wished to determine the prevalence of each health indicator from a national sample that closely matched our sample on key sociodemographic factors related to health. We first restricted each national data set to employed women aged 18 to 64 who had at least a high school education or general education diploma (GED), characteristics that matched our sample. After applying this restriction, we found sociodemographic differences between our sample and the national samples (Table 1). We then used direct adjustment procedures (14,15) to adjust the prevalence of each health indicator in the national data according to the distribution of our survey participants across strata of age, education, race/ethnicity, and marital status (Table 1). To adjust these national prevalence estimates, we used the survey commands in Stata/SE (version 12, StataCorp LP, College Station, Texas) to take into account the sample weights and variance estimates based on the complex sampling design of these surveys.

**Table 1:** Sociodemographic Characteristics of Pennsylvania Head Start Staff Survey Participants in 2012 and National Reference Populations in 2011

Characteristic	Head Start Staff (N = 2,122)		NHIS 2011 (N = 9,118)		BRFSS 2011 (N = 123,699)	
	n^a^	% (95% CI)	n^b^	% (95% CI)	n^c^	% (95% CI)
**Age, y**						
18–29	366	17.8 (16.1–19.4)	2,324	26.8 (25.4–28.1)	13,825	22.0 (21.4–22.6)
30–39	546	26.5 (24.6–28.4)	2,162	21.3 (20.4–22.3)	22,528	22.2 (21.8–22.7)
40–49	497	24.1 (22.3–26.0)	2,069	23.0 (21.9–24.0)	31,484	25.2 (24.8–25.7)
≥50	651	31.6 (29.6–33.6)	2,563	28.9 (27.8–30.1)	55,862	30.6 (30.1–31.0)
**Highest education level**						
High school or GED	421	20.0 (18.3–21.7)	2,098	23.3 (22.2–24.4)	30,106	27.0 (26.5–27.5)
Some college	415	19.7 (18.0–21.4)	3,552	38.4 (37.0–39.7)	37,547	36.3 (35.8–36.9)
Bachelor’s degree or higher	1,270	60.3 (58.2–62.4)	3,468	38.3 (36.9–39.8)	56,046	36.7 (36.1–37.2)
**Race/ethnicity**						
Non-Hispanic white	1,804	85.7 (84.2–87.2)	5,535	70.5 (69.2–71.8)	96,375	69.7 (69.1–70.2)
Non-Hispanic black	126	6.0 (5.0–7.0)	1,532	12.8 (11.9–13.7)	11,365	12.5 (12.1–12.9)
Non-Hispanic other	53	2.5 (1.8–3.1)	737	6.2 (5.6–6.8)	7,036	7.2 (6.9–7.6)
Hispanic (any race)	123	5.8 (4.8–6.8)	1,314	10.5 (9.8–11.2)	8,143	10.6 (10.2–11.0)
**Relationship status**						
Married	1,310	62.4 (60.3–64.4)	3,902	52.9 (51.6–54.2)	71,634	52.6 (52.1–53.2)
Not married	791	37.6 (35.6–39.7)	5,195	47.1 (45.8–48.4)	51,598	47.4 (46.8–47.9)

Abbreviations: NHIS, National Health Interview Survey; BRFSS, Behavioral Risk Factor Surveillance System; CI, confidence interval; GED, general educational diploma.

a Data were missing for age (n = 62), race/ethnicity (n = 16), relationship status (n = 21), and education level (n = 16).

b Unweighted n’s and weighted percentages among employed women aged 18 to 64 whose highest education level was at least high school or GED. Data were missing on relationship status (n = 21).

c Unweighted n’s and weighted percentages among employed women aged 18 to 64 whose highest education level was at least high school or GED. Data were missing on race/ethnicity (n = 780) and relationship status (n = 467).

## Results

Among survey participants, 55.7% were aged 40 years or older, 60.3% finished college, 85.7% were non-Hispanic white, and 62.4% were married (Table 1). By job category, 48.8% were teachers (lead or assistant); 14.4%, home-based visitors; 15.0%, family support staff; and 21.8%, managers.

All 6 physical health conditions were more common among Head Start staff than among the national sample (Table 2). Although 23.6% of Head Start staff reported having none of the 6 physical health conditions, 21.8% reported having 3 or more (Table 2). In the national sample, 35.1% reported having none of the 6 physical health conditions, and 12.6% reported having 3 or more. The prevalence of diagnosed depression was higher among Head Start staff than among the national sample. Based on responses to the CES-D, 24.4% of Head Start staff were depressed, and of these, 55.8% reported not ever receiving a diagnosis of depression. We found depression indicated by CES-D or diagnosis in 37.0% of Head Start staff.

**Table 2:** Prevalence of Heath Indicators Among Pennsylvania Head Start Staff Survey Participants (N = 2,122) in 2012 and a National Reference Population in 2011^a^

Health Indicator	Head Start Staff, % (95% CI)	National Reference, % (95% CI)	Difference, % (95% CI)
**Physical health**			
Severe headache or migraine	32.2 (30.2–34.2)	21.6 (20.4–22.8)	10.6 (8.3–12.9)
Lower back pain	36.9 (34.9–40.0)	23.9 (22.6–25.1)	13.0 (10.6–15.4)
Obesity	37.1 (34.9–39.3)	27.3 (26.0–28.6)	9.8 (7.3–12.3)
Asthma	18.7 (17.0–20.3)	13.6 (12.5–14.7)	5.0 (3.1–7.0)
High blood pressure	22.3 (20.5–24.0)	18.1 (17.0–19.2)	4.1 (2.0–6.2)
Diabetes or prediabetes	11.9 (10.5–13.3)	7.8 (7.1–8.6)	4.1 (2.5–5.6)
≥3 Physical health conditions	21.8 (20.0–23.6)	12.6 (11.7–13.5)	9.2 (7.3–11.2)
**Mental health**			
Depression diagnosed by health professional	23.5 (21.7–25.3)	17.6 (17.1–18.0)	5.9 (4.0–7.8)
**Health-related quality of life**			
Fair or poor health status	14.6 (13.1–16.1)	5.1 (4.5–5.6)	9.5 (7.9–11.1)
Frequent physically unhealthy days (≥14 d/mo)	10.1 (8.8–11.4)	5.9 (5.6–6.1)	4.2 (2.9–5.5)
Frequent mentally unhealthy days (≥14 d/mo)	18.0 (16.4–19.7)	9.5 (9.2–9.8)	8.5 (6.8–10.2)
Frequent physically or mentally unhealthy days (≥14 d/mo)	28.3 (26.3–30.2)	14.5 (14.1–14.9)	13.7 (11.8–15.7)
Work absences due to illness (≥10 d/y)	8.6 (7.4–9.8)	7.4 (6.7–8.2)	1.2 (−0.2 to 2.6)
**Health care access**			
Has personal doctor	96.5 (95.7–97.3)	86.4 (86.0–86.8)	10.1 (9.2–11.0)
Has health insurance	96.4 (95.6–97.2)	89.5 (88.8–90.3)	6.9 (5.8–8.0)
Has visited dentist in last 12 months	76.4 (74.6–78.3)	76.2 (75.0–77.5)	0.2 (−2.0 to 2.4)

Abbreviation: CI, confidence interval.

a All national estimates were obtained from the National Health Interview Survey (NHIS) 2011, except for the following: diagnosed depression; number of physically unhealthy days, mentally unhealthy days, and physically or mentally unhealthy days; and access to a personal doctor. Data on these latter items were obtained from the Behavioral Risk Factor Surveillance System (BRFSS) 2011. Weighted percentages were from a restricted sample from either NHIS (n = 9,118) or BRFSS (n = 123,699). Both national samples were restricted to employed women aged 18 to 64 years whose highest education level was at least high school or a general education diploma; to match the Head Start sample, percentages were further adjusted for age, race/ethnicity, education level, and marital status.

Fair or poor health status was almost 3 times more common among Head Start staff than among the national sample (Table 2). Among Head Start staff, having frequent mentally unhealthy days was almost twice as common as having frequent physically unhealthy days. Although the prevalence of frequent unhealthy days was higher among Head Start staff than among the national sample, the prevalence of 10 or more days per year of work absence due to illness was not significantly higher. Among Head Start staff, 48.5% of those who reported frequent physically unhealthy days also reported frequent mentally unhealthy days, compared with 33.0% in the national sample. Among Head Start staff who reported frequent physically unhealthy days, 24.2% reported 10 or more days per year of work absences due to illness; among those who reported frequent mentally unhealthy days, 12.9% had 10 or more days per year of work absences due to illness. When we further restricted the national samples to those with an annual household income below $50,000, which is in the upper salary range reported for Head Start managers in Pennsylvania, the findings shown in Table 2 did not change.

Among Head Start staff, the prevalence of frequent mentally unhealthy days, frequent physically unhealthy days, and 10 or more days per year of work absence due to illness was greater among those with 3 or more physical health conditions than among those with fewer than 3 conditions (Table 3). The prevalence of frequent unhealthy days and work absences was also greater among Head Start staff who were classified as depressed according to the CES-D than among those who were not so classified (Table 3). Having 3 or more physical health conditions was more common among those with CES-D–defined depression than among those without it (31.5% vs 18.8%; odds ratio, 1.98; 95% confidence interval, 1.58–2.48).

**Table 3:** Prevalence of Unhealthy Days and Absences, by Number of Physical Conditions and CES-D Score Among Pennsylvania Head Start Staff, 2012

Health Indicator	Number of Physical Conditions^a^			CES-D Score^b^		
	≥3 Conditions, % (n = 459)	<3 Conditions, % (n = 1,645)	Odds Ratio (95% Confidence Interval)	Score ≥16, % (n = 513)	Score <16, % (n = 1,589)	Odds Ratio (95% Confidence Interval)
≥14 Physically unhealthy days/month	19.9	7.2	3.18 (2.37–4.29)	18.1	7.3	2.78 (2.07–3.74)
≥14 Mentally unhealthy days/month	26.6	15.6	1.96 (1.53–2.51)	51.7	7.2	13.83 (10.66–17.94)
Absent from work ≥10 days/year due to illness	16.0	6.6	2.72 (1.98–3.73)	12.8	7.3	1.87 (1.36–2.58)

Abbreviations: CES-D, Center for Epidemiologic Studies Depression Scale.

a Number of physical conditions among the following 6: obesity (body mass index ≥30 kg/m^2^ based on self-reported height and weight), diagnosed asthma, diagnosed hypertension, diagnosed diabetes/prediabetes, severe headache/migraine in last 3 months, and lower back pain in last 3 months. Data on the physical health conditions score were missing for 18 of 2,122 participants.

b Data on the CES-D were missing for 20 of 2,122 participants.

Nearly all Head Start staff (>96%) reported having health insurance and a personal doctor, a greater percentage than in the national sample. Almost 25% of Head Start staff had not visited a dentist in the previous year, which was also comparable with findings for the national sample.

## Discussion

Among women working in Head Start and Early Head Start Programs in Pennsylvania, the prevalence of physical and mental health conditions, fair or poor health status, and frequent mentally and physically unhealthy days were higher than among employed women in a national sample of women of similar age, education, race/ethnicity, and marital status. We identified only 1 US study on the prevalence of physical health indicators within a population-based sample of staff working in early childhood care and education settings (16). In that 1993 study of 446 early childhood workers in Wisconsin, the prevalences of fair or poor health, back pain, and headache were lower than were those in our study. However, the data are not directly comparable because the Wisconsin survey used different questions. In a more recent survey of 168 early childhood workers in Wellington, New Zealand, the investigators used the same questions used in the Wisconsin survey and showed lower prevalences of fair or poor health, back pain, and headache than those found in our study (17). In our study, 24.4% of Head Start staff had clinically significant depressive symptoms (CES-D score ≥16). A national study of early childhood care and education staff used the same measure and found a prevalence of 9.4% (18). The difference may have resulted from our survey being anonymous or our sample having only staff working in Head Start and Early Head Start.

Our study had several limitations. The survey respondents may not be representative of all Head Start and Early Head Start staff working in Pennsylvania. Because some programs in the state did not participate in the survey, staff working in urban programs, particularly in Philadelphia, were underrepresented. Within the 66 participating programs, the education level and racial/ethnic composition of the participating staff were similar to those of the whole staff. However, nonresponse bias due to a healthy-volunteer effect within participating programs may have led to an underestimate of the prevalence of health indicators (19). Response rates may have been higher if we had identified individual staff and provided them reminders and monetary incentives. However, we felt that an anonymous survey would provide greater assurance of confidentiality and, therefore, increase participation and reduce social desirability bias. Finally, the differences in the prevalence of health indicators between Head Start staff and the national samples may have been overestimated because we did not collect data on household income and size and could not adjust for it. However, all salaries in Head Start are relatively low, with the average Head Start teacher in Pennsylvania earning approximately $26,000 a year. 

The overall goal of Head Start and Early Head Start is to increase the school readiness of economically and socially disadvantaged children. Achieving this goal depends on the functioning of the staff, but the staff must be well — both physically and mentally — to optimally use their skills and training. The healthy development of children in all early childhood care and education settings relies not only on adults being physically and emotionally engaged but also on being consistently present at work (20). Almost 30% of Head Start staff reported feeling physically or mentally unhealthy more than 2 weeks in the previous month, and almost 10% reported missing 2 or more weeks of work in the previous year due to illness.

The high prevalence of depressive symptoms found in this study and its potential impacts on children in Head Start and Early Head Start requires further study. Although research finds that maternal depression can adversely impact young children (21,22), similar evidence does not exist about the impact of depression among early childhood educators on children’s outcomes. Symptoms of depression in early childhood staff are associated with lower sensitivity and increased withdrawal in adult–child interactions (18). Staff’s symptoms of depression may also interfere with the development of healthy self-regulation in children, which could affect school readiness (23).

In addition, the physical health of Head Start staff is important, especially given the increasing attention in early childhood care and education programs to the role of movement in children’s learning and health (24). This emphasis on movement reflects a long-standing appreciation of how young children learn through movement (25), and the potential contribution of movement to preventing early childhood obesity. Obesity and its comorbidities, along with back pain and headache, may impair the ability of Head Start staff to model healthy movement and engage children by moving with them. To address childhood obesity, the Office of Head Start implemented a nationwide program enhancement (I Am Moving, I Am Learning) that includes activities to improve the diet and physical activity behaviors of the Head Start staff (26). In child care centers outside Head Start, similar efforts with staff have been made (27).

Improving child outcomes in Head Start and Early Head Start may require more attention to the health and well-being of the staff. Some promising approaches to improving staff wellness through professional development and staff training models include mindfulness-based stress-reduction techniques (28). Such approaches have already been applied in other professions, such as health care, public safety, and social work, in which high demands are placed on staff who must be physically and mentally well to serve others (29,30). Bibliotherapy, the use of books or other reading materials for help in solving personal problems, is another promising approach to addressing staff wellness because it is low-cost and avoids stigma (31). In the area of physical health, worksite models for teachers are emerging as a component of school-based obesity prevention programs (32).

In Head Start and Early Head Start, as well as in other early childhood care and education programs, staff are the necessary link between program content and children’s outcomes. However, our data suggest that the women working with children in Head Start and Early Head Start have poorer physical and mental health than do US women with similar sociodemographic characteristics. More investments may be required to support the health and well-being of those adults to whom the public entrusts children’s development and learning outside the home.
